# Principles of Surgery

**Published:** 1896-02

**Authors:** 


Principles of Surgery. By N. Senn, M. D., Ph. D., LL. D., Professor of Practice of Surgery and Clinical Surgery in Rush Medical College, Chicago; Professor of Surgery in the Chicago Polyclinic; Attending Surgeon to the Presbyterian Hospital; Surgeon-in-Chief to St. Joseph's Hospital; Ex-President American Surgical Association, etc. Second Edition. Thoroughly Revised. Illustrated with 178 Wood Engravings and five (5) Colored Plates. Royal Octavo, pages xvi, 656. Extra Cloth, $4.50 net; Sheep or Half-Russia, $5.50 net. Philadelphia: The F. A. Davis Co., Publishers, 1914 and 1916 Cherry Street.

This work has been brought to the attention of our readers before, having been noticed in the number for October, 1891, at page 407. The volume now before us is the second edition, and is much enlarged and otherwise improved. It is different from other works on surgery noticed from time to time in these pages, in that it deals but little with operative procedures and confines itself to the physiological and pathological processes involved in surgical injuries and surgical diseases. The book is actually what its names implies, a treatise upon the *principles* of surgery. In the domain which it specially occupies it may be truly called a beautiful book. He must be an accomplished physician who has clearly in his mind all the teachings of this elegant volume. The reviewer does not pretend to discuss this work, nor to criticise or elaborate the views of the author. That would be too extensive a task. A fair idea of the book may be had by a glance at its table of contents. Here are some of the headings of the chapters; Regeneration of Different Tissues; Inflammation; Pathogenic Bacteria; Necrosis; Suppuration; Ulceration and Fistula; Suppurative Osteo-myelitis; Septicæmia; Pyæmia; Erysipelas; Tetanus; Hydrophobia; Surgical Tuberculosis; Actinomycosis Hominis; Anthrax, Glanders.

It will be seen from this that the book treats of the intimate nature of these various processes, an understanding of which by our latest lights is so necessary to the management of such cases.

